# Supplementary material for: Patterns of Intron Gain and Loss in Fungi
Source: PLoS Biol. 2004 Nov 30;2(12):e422. doi: 10.1371/journal.pbio.0020422 (PMC532390; doi:10.1371/journal.pbio.0020422)
Supplement: Table S1 — Also available at http://genes.mit.edu/NielsenEtAl/. (4.3 MB ZIP). [file pbio.0020422.st001.zip › NielsenEtAl/html/966.html]

AN5020.1.NCU07173.1.MG10676.1.FG04483.1


```
 CLUSTAL W (1.82) Multiple Sequence Alignments - Introns Inserted


Sequence 1: MG10676.1	186 aa
Sequence 2: FG04483.1	186 aa
Sequence 3: AN5020.1	185 aa
Sequence 4: NCU07173.1	184 aa
Alignment Length: 188 aa
Number Identitical Residues: 120 aa
Alignment Score (without introns) 5259


MG10676.1 	MGGQLSK-MMGKIFGSKEMRLLMLGLDAAGKTT~ILYKLKLG-QDVTTIPTVGFNVETVT
NCU07173.1	MGNVMSLGFMAKLFGTKEVRVLMLGLDAAGKTT~ILYKLKLQ-DHVTTIPTVGFNVETVT
FG04483.1 	MGGQFSK-MMGKIFGSKEMRLLMLGLDAAGKTT~ILYKLKLG-QDVTTIPTVGFNVETVT
AN5020.1  	MGGSVSK-IMGKIFGTKEMRILMLGLDAAGKTT1ILYKLKLTNQDVTTIPTVGFNVESVT
          	**. .*  :*.*:**:**:*:************ ******* .:.************:**

MG10676.1 	YKNVKFNVWDVGGQDKIRPLWRHYFSG1TQGLIFVIDSSDRKRMDEARQELHRIINDREM
NCU07173.1	LRNIKFNIWDVGGQDKIRPLWRHYYSG~TQGLIFVVDSADHNRIDEARVELHRIINDREM
FG04483.1 	YKNVKFNVWDVGGQDKIRPLWRHYFSG1TQGLIFVIDSSDKNRMEEARQELHRIINDREM
AN5020.1  	YKNVKFNVWDVGGQDKIRPLWRHYYSG1TQGLIFVVDSSDTARMDEARSELHKIINDREM
          	 :*:***:****************:** *******:**:*  *::*** ***:*******

MG10676.1 	KDSLLLVFANKQDLENA1MKPQEVTEALQLSKLKDKIWYVVPSCATTGEGLLEGL~AWLS
NCU07173.1	KDCLLLVFANKQDIAGA1MTPQEVTEKLQLNKLKDRIYAVQPTIAIDKAGLQEGF~NWLS
FG04483.1 	KDSLLLVFANKQDLAEA1MSPQEVTEALQLSKLKDKVWYVVPSCATTGEGLLEGL~AWLS
AN5020.1  	KDALLLVFANKQDVPGH1LSPDEVISALKLHSLKDKTWYVAPSVATEGTGIFEGL0AWLS
          	**.**********:    :.*:** . *:* .***: : * *: *    *: **:  ***

MG10676.1 	NNVKAPPAPAKK
NCU07173.1	DNMKAAQKQ---
FG04483.1 	NNVKAPPTPAKK
AN5020.1  	NNVKVQ-QPQK-
          	:*:*.     .
```
